# Complement activation products in tears of dry eye and meibomian gland dysfunction

**DOI:** 10.1038/s41598-023-46634-7

**Published:** 2024-01-02

**Authors:** Hiroki Maehara, Koki Norikawa, Keiichiro Tanaka, Yutaka Kato, Akihito Kasai, Ryo Mukai, Tomoko Omori, Takeshi Machida, Hideharu Sekine, Tetsuju Sekiryu

**Affiliations:** 1 Department of Ophthalmology, Fukushima Medical University School of Medicine, Hikarigaoka 1, Fukushima, Fukushima 960–1247 Japan; 2 Department of Immunology, Fukushima Medical University School of Medicine, Fukushima, Japan

**Keywords:** Eye diseases, Molecular medicine

## Abstract

Sixty-seven patients (38 woman; median age, 69 years) were enrolled to assess complement activation products (CAPs) in tear fluid with/without dry eye (DE) and with/without meibomian gland dysfunction (MGD). Patients were divided into four groups based on the presence/absence of DE and MGD: group DM had both DE and MGD, group DN had DE without MGD, group NM had MGD without DE, and group NN had neither DE nor MGD. The levels of C3a and C5a in the collected tears were analyzed using a cytometric bead array. The C3a concentrations in the DM, DN, NM, and NN groups were 2326 pg/ml, 1411 pg/ml, 1821 pg/ml, and 978 pg/ml, respectively. The C5a concentrations in the DM, DN, NM, and NN groups were 24.7 pg/ml, 15.3 pg/ml, 24.1 pg/ml, and 12.9 pg/ml, respectively. The concentrations of C3a and C5a in the DM and NM groups were significantly higher than in the NN group (*P* < 0.05 for both comparisons). The CAPs in the tear fluid in MGD and DE increased. Local dysregulation of the innate immune system can be associated with the development of MGD and DE in elderly patients.

## Introduction

Dry eye (DE) is believed to cause ocular surface inflammation, damage, and sensory nerve abnormalities, and subjective symptoms such as eye dryness and eye discomfort, instability of the tear fluid layer, hyperosmolarity, and inflammation of the ocular surface are considered to be the causes^1,2^. Meibomian gland dysfunction (MGD) increases in the elderly population. Recent reports have suggested that DE also increases in older populations^3–5^ and is a cause of DE with increased evaporation, with a prevalence rate as high as 32.9%. Decreased lipid secretion function may cause instability of the tear fluid layer, ocular inflammation, and ocular surface diseases^3,6,7^.

Although inflammation of the ocular surface causes DE and MGD or is a consequence of these diseases, the reason is not yet understood. Many reports on proteins in tear fluid in DE have been published. It is known that interleukin (IL)-6 in tear fluid is elevated in DE and that IL-6 is produced by inflamed epithelial cells^8,9^. In addition to IL-6, IL-1β, IL-8, IL-10, interferon-γ and tumor necrosis factor-α also are elevated in the tear fluid^9^.

**Figure 1:**
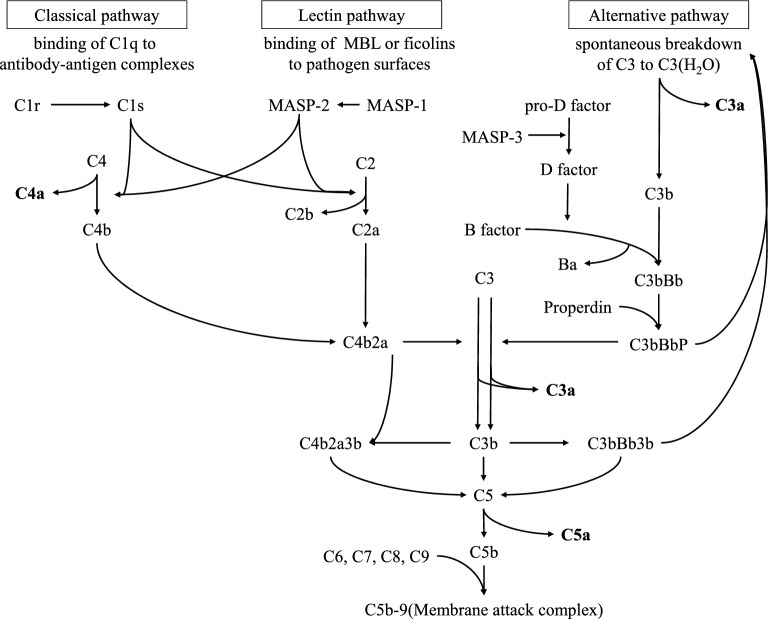
Complement pathway. The complement pathway is activated through one of the three pathways: the classical pathway, lectin pathway, or alternative pathway. It activates C3 through the formation of C4b2a or C3bBb, which are C3 convertases. The classical pathway and lectin pathway are connected to the alternative pathway through the production of C3b, which amplifies C3 activation. When C4b2a3b or C3bBb3b, which are C5 convertases, are formed, C5 is activated, leading to the formation of C5b-9 (membrane attack complex). (MBL: mannan-binding lectin).

Cytokines can regulate complement activation and modulate inflammatory responses induced by complement pathway. Cytokines and complement pathway are closely related in the regulation of the immune system and immune responses. There are three complement pathways: the classical pathway, lectin pathway, and alternative pathway. The complement pathway plays an important role in many acute inflammatory processes and host defense^10^ (Fig. 1). C3a is a recognized mediator of the inflammatory response that affects polymorphonuclear leukocytes, vasoconstriction, and leakage^11^. C3a is a recognized mediator of inflammatory responses and is credited with increasing vascular permeability^11^. C5a activates basophils, monocytes, and neutrophils, increasing vascular permeability^12^. In ophthalmology, it had been reported that the levels of complement activation products (CAPs) in the ocular tissue of mice increase with aging^13^. Indeed, previous reports had been demonstrated that the complement pathway was functional in the cornea, and there was a possibility of complement activation product formation in tears as well^14^. Furthermore, the complement pathway is activated in age-related macular degeneration^15–17^, bacterial keratitis^18^, and allergic conjunctivitis^19^. C3a in tear fluid is elevated in vernal catarrh, viral keratitis, and Sjögren's syndrome^20^ and may be associated with DE^21^. We have reported an increase in CAPs in tear fluid after ophthalmic surgery, indicating that the CAPs in tear fluid rise due to surgical invasion and inflammation^22^. This paper reports that ophthalmic surgery performed on a dry eye patient results in elevated levels of CAPs in the tear fluid for an extended period after the surgery. It was observed that inflammatory reactions continue on the ocular surface for a prolonged duration in cases of tear-decreased eyes. Although many studies have reported biochemical changes in the tear fluid and oil layer, few have investigated CAPs in the tear fluid of patients with DE and MGD. Therefore, studies were undertaken to investigate the association between CAPs in tears and the pathogenesis of age-related conditions such as DE and MGD, which are known to have an increased prevalence with aging.

## Materials and methods

### Patients

Patients were those who visited Fukushima Medical University Hospital and had not been treated for DE and MGD.

### Ethics statement

The Institutional Review Board of Fukushima Medical University, Fukushima, Japan, approved the study protocol (ID: 2021-076). The research was conducted in accordance with the tenets of the Declaration of Helsinki, and all patients provided written informed consent after they received a detailed explanation of the study protocols and possible consequences associated with participation.

### Ocular surface evaluation

The ocular surface parameters were evaluated in the following order: lower tear meniscus height (TMH), fluorescein break-up time (FBUT), ocular surface disease index (OSDI), and Schirmer I test. About 10 min after the fluorescein staining, the Schirmer I test was performed without anesthesia. The TMH measured the lower lacrimal meniscus using anterior optical coherence tomography (CASIAII, Tomey Corp., Nagoya, Japan)^23^. Two investigators (HM, KN) measured the lower TMH manually, and the average of the two measurements was recorded. The FBUT were measured based on the Asian and Japanese DE diagnostic criteria^2,24^ and the tear film and ocular surface (TFOS) DEWS II DE diagnostic procedure^1^. The diagnosis of MGD was based on the Japanese diagnostic criteria and the TFOS International Workshop on Meibomian Gland Dysfunction and Dry Eye Workshop 2.0^1,25^. OSDI was heard as previously reported^26^.

The exclusion criteria included a history of ophthalmic surgery; the presence of severe conjunctival chalasis, superior limbic keratoconjunctivitis, lid-wiper epitheliopathy, pterygium, or ocular surface diseases that could affect the evaluation of the FBUT; chronic use of DE drops or glaucoma eye drops; the presence of diseases affecting tear fluid such as type 2 diabetes, rheumatoid arthritis, and Sjögren's syndrome; and other factors considered inappropriate for inclusion such as inflammatory diseases of the ocular surface.

### Subjects and environmental conditions

Sixty-seven patients (38 women; median age, 69 years) were enrolled in this cross-sectional study. MGD and dry eye are known to increase with age, with previously reported rates of 41.9% for patients in their 60 s, 48.4% for patients in their 70 s, and 63.9% for patients in their 80 s^3^. To avoid case bias, patients were randomly selected from an age-matched group of patients who visited our hospital. DE and MGD were assessed by referring to Japanese and TFOS guidelines and evaluated by three ophthalmologists (H.M, K.N, K.T)^1,2,24,25^. In cases where there was a disagreement among the three experts, the diagnosis with the majority of votes was considered. Patients with DE and MGD (n = 20, 20 eyes, 14 women) were designated as the DM group, and those with DE and no MGD (n = 19, 19 eyes, 15 women), no DE and MGD (n = 14, 14 eyes, 10 women), and no DE and no MGD (n = 14, 5 women) were designated, respectively, as the DN, NM, and NN groups. The NN group was the control group. All examinations were performed under the environmental conditions of an average temperature of 23.9 ± 0.51 °C (range: 23–25 °C) and an average humidity of 52.6 ± 5.23% (range: 45–61%).

### Tear fluid collection and measurements

The tear fluid was collected non-invasively during the ocular surface evaluation as reported previously^27^. The same investigator (HM) collected the tear fluid in all cases. To avoid the effect of eye drops on the ocular surface measurements, participants were instructed not to use any eye drops on the day of tear fluid sample collection. The patient lay on his/her back on the bed and the clinician used a micropipette (Drummond Scientific, Broomall, PA, USA) to collect 2 μl of tear fluid from the external eyelid margins. The collected tear fluid was immediately stored at − 80 °C until analysis. The collected tears were measured using the BD CBA Human Anaphylatoxin Kit (BD Biosciences, San Jose, CA, USA) for CAPs (C3a, C4a, and C5a). The CAPs in the tear fluid were measured according to the previous reports and the manufacturer's instructions^17,22^.

### Statistical analysis

The Dunnett and Steel–Dwass tests were used to compare measurements to the NN group. Correlation coefficients were performed to identify the association of changes in ocular surface parameters. *P* < 0.05 was considered statistically significant. Statistical analyses were performed using JMP16 software (SAS Institute, Cary, NC, USA). The sample size was determined using a "Sample Size Calculator" with an alpha of 5% and a power (1-β) of 0.80. The sample size was calculated to be 13 in this study. Cases with any missing clinical data were excluded from the analysis in this study. Likewise, a student's t-test was performed for sensitivity analysis, and similar results were obtained.

## Results

### CAPs in tears

The C3a (pg/ml), C4a (pg/ml), and C5a (pg/ml) levels were 978 (interquartile range [IQR] 390–1396) (pg/ml), 441 (IQR 384–571) (pg/ml), and 12.9 (IQR 10.9–19.4) (pg/ml) in the NN group, and 1673 (IQR 1075–4002) (pg/ml), 534 (IQR 299–845) (pg/ml), and 21.3 (IQR 15.7–28.5) (pg/ml) in the other groups combined, respectively. Since there were significant differences in the concentrations of CAPs between the NN and all other groups, comparisons were also made with the DM, DN, and NM groups. Significant differences were seen between the NN group and the other groups in the C3a and C5a levels (*P* = 0.0025 and *P* = 0.0010, respectively), but the C4a level did not differ significantly (*P* = 0.41).

**Figure 2:**
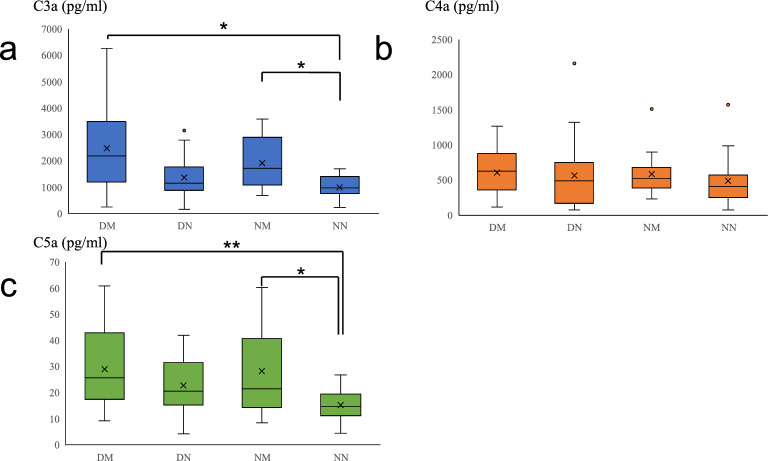
Complement activation products in tears in each group. (**a**) C3a levels in tears in each group; (**b**) C4a levels in tears in each group; **c** C5a levels in tears in each group. DM, dry eye and meibomian gland dysfunction group; DN, dry eye and non-meibomian gland dysfunction group; ND, non-dry eye and meibomian gland dysfunction group; NN, non-dry eye and non-meibomian gland dysfunction group. **P < 0.01, *P < 0.05.

Figure 2 shows the CAP levels in the tears in each group. The C3a (pg/ml) levels in the DM, DN, NM, and NN groups were 2326 (IQR 1223–4012) (pg/ml), 1411 (IQR 891–1919) (pg/ml), 1821 (IQR 1068–2890) (pg/ml), and 978 (IQR 390–1396) (pg/ml), respectively. The significance levels between each group were as follows: DM versus NN, *P* = 0.0013; DN versus NN, *P* = 0.38; and NM versus NN, *P* = 0.032.

The C4a (pg/ml) levels in the DM, DN, NM, and NN groups were 622 (IQR 188–898) (pg/ml), 493 (IQR 171–770) (pg/ml), 534 (IQR 413–865) (pg/ml), and 441 (IQR 384–571) (pg/ml), respectively. No significant differences were seen between each group as follows: DM versus NN, *P* = 0.79; DN versus NN, *P* = 0.99; and NM versus NN, *P* = 0.76.

The C5a (pg/ml) levels in the DM, DN, NM, and NN groups were 24.8 (IQR 15.8–43.4) (pg/ml), 20.8 (IQR 15.3–36.3) (pg/ml), 24.1 (IQR 15.6–41.7) (pg/ml), and 12.9 (IQR 10.9–19.4) (pg/ml), respectively. Significant differences were seen between each group as follows: DM versus NN, *P* < 0.001; DN versus NN, *P* = 0.069; and NM versus NN, *P* = 0.0015.

### Ocular surface parameters

Table 1 summarizes the clinical data in the four groups. The TMH (μm) of the DM, DN, NM, and NN groups were 210 (IQR 180–231) (μm), 204 (IQR 177–267) (μm), 251 (IQR 224–275) (μm), and 222 (IQR 206–248) (μm), respectively. No significant differences were seen compared to the NN group: DM versus NN, *P* = 0.66; DN versus NN, *P* = 0.67; and NM versus NN, *P* = 0.27.

**Table 1:** Ocular surface parameters and complement actvation products in tears.

		DE and MGD group	*P* value*	DE and non-MGD group	*P* value*	Non-DE and MGD group	*P* value*	Non-DE and non-MGD group
Male / Female		6 / 14	N/A	4 / 15	N/A	10 / 4	N/A	9 / 5
Age	[Median (IQR)]	69 (65–73)	0.66	68 (65–74)	0.95	71 (67–74)	0.51	68 (62–72)
TMH (μm)	[Median (IQR)]	210 (180–231)	0.66	204 (177–267)	0.67	251 (224–275)	0.27	222 (206–248)
FBUT (sec)	[Median (IQR)]	1 (0–1.75)	< 0.001	1.7 (0.8–2)	< 0.001	5.8 (5–7.8)	0.46	6.8 (6–7.8)
OSDI	[Median (IQR)]	11.4 (4.2–16.7)	0.025	10.0 (4.2–20.5)	0.027	4.4 (1.6–6.3)	0.98	4.2 (1.5–6.3)
Schirmer I test (mm)	[Median (IQR)]	5 (4–13)	0.0014	5 (4–15)	0.026	13.5 (10–20)	0.67	15.5 (14.5–18.3)
C3a (pg/ml)	[Median (IQR)]	2326 (1223–4012)	0.0013	1411 (891–1919)	0.38	1821 (1068–2890)	0.032	978 (390–1396)
C4a (pg/ml)	[Median (IQR)]	622 (188–898)	0.79	493 (171–770)	0.99	534 (413–865)	0.76	441 (384–571)
C5a (pg/ml)	[Median (IQR)]	24.7 (15.7–43.4)	< 0.001	15.3 (20.7–36.3)	0.069	24.1 (15.6–41.7)	0.0015	12.9 (10.9–19.4)

DE, Dry eye; MGD, Meibomian grand dysfunction; IQR, Interquartile range; TMH, Tear meniscus height; FBUT, Fluorescein breakup-time; OSDI, Ocular surface disease index.

*versus non-DE and non-MGD group, ANOVA.

The FBUTs (seconds) of the DM, DN, NM, and NN groups were 1 (IQR 0–1.8) (seconds), 1.7 (IQR 0.8–2) (seconds), 5.8 (IQR 5–7.8) (seconds), and 5.8 (IQR 5–7.8) (seconds), respectively. Significant differences were seen compared to the NN group as follows: DM versus NN, *P* = 0.0014; DN versus NN, *P* = 0.027; and NM versus NN, *P* = 0.67.

The OSDI of the DM, DN, NM, and NN groups were 11.4 (IQR 4.2–16.7), 10.0 (IQR 4.2–20.5), 4.4 (IQR 1.6–6.3), and 4.2 (IQR 1.5–6.3), respectively. Significant differences were seen compared to the NN group as follows: DM versus NN, *P* = 0.025; DN versus NN, *P* = 0.027; and NM versus NN, *P* = 0.98.

The Schirmer I test results (mm) of the DM, DN, NM, and NN groups were 5 (IQR 4–13) (mm), 5 (IQR 4–15) (mm), 13.5 (IQR 10–20) (mm), and 15.5 (IQR 14.5–18.3) (mm), respectively. Significant differences were seen compared to the NN group as follows: DM versus NN, *P* = 0.0014; DN versus NN, *P* = 0.027; and NM versus NN, *P* = 0.67.

### Relationships between ocular surface parameters and CAPs in tears

To examine the relationship between CAPs and ocular surface parameters, we first analyzed the correlation between the disease (DM, NM, ND) and NN groups.

A negative correlation between C3a and Schirmer I test was seen in the disease groups (r^2^ = − 0.44, *P* = 0.0010). When univariate and multiple analyses were performed, in the NM group, the C3a level was significantly correlated with TMH, FBUT, and Schirmer I test (*P* = 0.0020, *P* = 0.020, *P* = 0.024, respectively), and the C5a level was correlated with the FBUT (*P* = 0.027) (Table 2). In the DM group, the concentrations of C3a and C5a were correlated with Schirmer I test in the DM group (*P* = 0.035, *P* = 0.042, respectively). OSDI was not correlated with the various CAPs in each group.

**Table 2:** Univariate and multivariate analysis of ocular surface parameters and C3a, C4a and C5a in each group.

Independent variables	C3a				C4a				C5a			
	Univariate analysis		Multivariate analysis		Univariateanalysis		Multivariate analysis		Univariateanalysis		Multivariate analysis	
	RC	*P* value	SRC	*P* value	RC	*P* value	SRC	*P* value	RC	*P* value	SRC	*P* value
Dry eye and meibomian gland dysfunction group												
TMH	− 0.38	0.10	− 0.27	0.23	− 0.45	0.054	− 0.18	0.42	− 0.36	0.12	0.36	0.16
FBUT	0.028	0.91	0.047	0.83	0.34	0.15	0.44	0.054	0.41	0.071	0.19	0.47
OSDI	− 0.27	0.25	0.12	0.61	0.15	0.54	0.31	0.18	0.084	0.72	0.18	0.42
Schirmer I test	− 0.53	0.016	− 0.48	0.035	− 0.28	0.24	− 0.39	0.082	− 0.34	0.14	− 0.56	0.042
Dry eye and non-meibomian gland dysfunction group												
TMH	0.22	0.41	0.36	0.16	0.15	0.56	0.26	0.35	0.043	0.87	0.14	0.64
FBUT	− 0.050	0.84	0.19	0.47	− 0.079	0.76	0.12	0.68	0.084	0.74	0.21	0.49
OSDI	− 0.066	0.79	0.053	0.86	− 0.34	0.16	− 0.27	0.27	− 0.034	0.89	0.15	0.54
Schirmer I test	− 0.44	0.064	− 0.56	0.054	− 0.29	0.24	− 0.43	0.14	− 0.11	0.65	− 0.26	0.39
Non-dry eye and meibomian gland dysfunction group												
TMH	0.59	0.026	0.68	0.0020	0.6	0.073	0.39	0.55	0.13	0.66	0.29	0.28
FBUT	− 0.32	0.26	− 0.45	0.020	0.15	0.62	0.013	0.96	− 0.58	0.031	− 0.65	0.027
OSDI	0.11	0.70	0.12	0.69	− 0.36	0.21	− 0.11	0.71	− 0.050	0.86	− 0.15	0.61
Schirmer I test	− 0.49	0.072	− 0.42	0.024	− 0.14	0.63	− 0.11	0.66	− 0.021	0.94	0.048	0.85
Non-dry eye and non-meibomian gland dysfunction group												
TMH	− 0.049	0.89	0.35	0.37	0.063	0.85	0.48	0.21	− 0.17	0.62	0.30	0.39
FBUT	− 0.23	0.43	− 0.77	0.072	− 0.40	0.16	− 0.60	0.13	− 0.40	0.15	− 0.69	0.073
OSDI	− 0.34	0.23	− 0.16	0.57	− 0.16	0.58	0.084	0.77	− 0.19	0.51	− 0.051	0.92
Schirmer I test	0.39	0.17	0.24	0.46	− 0.27	0.34	− 0.35	0.27	− 0.042	0.89	− 0.33	0.27

RC, Regression coefficient; SRC, Standardised regression coefficient; TMH, Tear meniscus height; FBUT, Fluorescein break-up time; OSDI, Ocular surface disease index.

## Discussion

The C3a and C5a levels were significantly higher in the DM and NM groups compared to the NN group. In the NM group, there was a significant correlation between the C3a levels and FBUT, Schirmer I test, and TMH. There also was a correlation between C3a and C5a levels and Schirmer I test in the DM group. To the best of our knowledge, this is the first study to identify a correlation between elevated CAP levels in tears and DE and/or MGD.

The C3a and C5a levels differed significantly between the NN group and all other groups. Furthermore, significant differences in the C3a and C5a levels were seen between the DM–NN and NM–NN groups but not between the DN–NN group. This suggested that the C3a and C5a level is elevated in MGD rather than DE. Since the C4a level was unchanged in DE and MGD, it is likely that the alternative complement pathway is activated in tear fluid in patients with DE and MGD. Previous reports have indicated that the levels of CAPs in the ocular tissue of mice increase with aging^13^. Additionally, it had demonstrated the functionality of the complement pathway in the cornea, suggesting the potential formation of CAPs in tears as well^14^. Because C3a and C5a enhance neutrophil activation and vascular permeability^28,29^, it can be inferred that this occurs in DE and MGD.

A comparison of TMH among the groups showed a higher value in the NM group, but the difference did not reach significance. In the NM group, increased C3a was associated with decreased Schirmer I test and shortened FBUT, while increased C3a was correlated positively with increased TMH. It has been reported that there is no difference in the TMH in MGD cases compared to normal cases and that there is no correlation between the cytokines in tear fluid and TMH in MGD cases^30^. Since MGD cases release a great deal of C3a into the tear fluid that causes inflammation, we assumed that the tear fluid volume was high to maintain ocular surface homeostasis. In the DE group, it is possible that the CAP concentration would be high because the tear volume was expected to be small. However, in the present study, a positive correlation was observed only between the TMH and C3a in the NM group, and there were no significant differences among the four groups. Therefore, the CAP concentration was not high because of the low TMH.

The FBUT was correlated negatively with C3a and C5a in the NM group. The complement system, a proteolytic cascade that mediates innate immunity and actively regulates the inflammatory response of DE, was correlated negatively with the FBUT as previously reported^30,31^. It has been reported that DE stimulates the production of inflammatory cytokines from the ocular surface epithelium, and the previous results showed a negative correlation between FBUT and IL-6^32^. In other words, the low volume of tear fluid on the ocular surface does not necessarily mean that the concentrations of IL-6 and other cytokines are low, suggesting that it may be the result of localized production that is elevated in disease-specific conditions. The negative correlation between CAP and MGD rather than DE in the present study suggests that CAP may be related more closely to MGD than DE.

As in previous studies, OSDI increased in MGD and DE. However, there was no observed correlation with CAPs. As discussed later, it is conceivable that a relationship may become evident when categorizing DE into subtypes, such as aqueous-deficient or increased-evaporation types. It is our belief that further investigations with an increased number of cases will be necessary in the future.

The intergroup comparison of the Schirmer I test results was significantly lower in the DM and DN groups than in the NN group. A negative correlation was observed between Schirmer I test and C3a, Schirmer I test and C5a in the DM group and Schirmer I test and C3a in the NM group. The C3a level was higher in MGD patients with decreased lacrimal secretion. In the presence of DE and MGD, the alternative pathway was considered to be enhanced. Previous studies have reported a negative correlation between the Schirmer I test and cytokines in tear fluid, and the same was true for C3a and C5a in the present study^32,33^. In the NM group, the positive correlation between the TMH and C3a was thought to result from the increased tear fluid volume to maintain ocular surface homeostasis in response to inflammation caused by MGD.

MGD is defined as “a chronic abnormality of meibomian glands characterized by terminal duct obstruction or qualitative or quantitative changes in the glandular secretion, which can result in alteration of the tear film, inflammation, ocular surface disease, and symptoms of eye irritation" by the Tear Film and Ocular Surface Society and is thought to induce inflammation such as posterior blepharitis and meibomian inflammation^25^. A reason for the elevated CAP in tear fluid in the groups with MGD in this study may be inadequate lipid supply, which may result in increased inflammation. A previous report showed that fatty acid uptake and de novo lipogenesis in the liver decreased as a result of a deficiency of factor D in rodents^34^, which suggested that complement components potentially regulate fatty metabolism. Factor D is required to generate the C3 convertase complex and is essential for producing C3a^35^. In this study, C3a was up-regulated, which suggested that factor D also should be co-activated in the MGD, which can modify lipid metabolism in MGD. Reduced lipid secretory function leads to decreased meibum secretion from the meibomian glands, causing hyperkeratosis of the ductal epithelium and consequently, the development of MGD^36^. This, in turn, leads to inflammation of the eyelids and lacrimal fluid.

Evidence suggests that DE stimulates the production of inflammatory cytokines from the ocular surface epithelium^37^. In this study, there were significant increases in the levels of C3a and C5a in tear fluid in the DM, DN and NM groups compared to the NN group, suggesting that the complement pathway in tear fluid may be activated in the presence of ocular surface disease. Since the CAP levels in tear fluid was the highest in the DM group and the CAP level was higher in the NM group than in the DN group, we considered that CAPs in tear fluid was strongly affected by MGD.

Inflammation is believed to be involved in MGD and DE. Inflammation is often treated in both diseases. Although cytokines in the tear fluid have been the target of treatment previously, we should also focus on CAPs in the tear fluid. Anti-complement drugs for paroxysmal nocturnal hemoglobinuria and thrombotic microangiopathy already exist and are effectively used in the real world to improve the prognoses of patients^38,39^. In MGD and DE, the treatment of the disease may change with the use of anti-complement or complement activating drugs. It is necessary to examine each type of MGD and DE to determine which findings most affect the CAP levels in the tear fluid and how the CAP levels change with treatment.

The correlation between ocular surface parameters and CAPs indicates that the tear concentrations may vary depending on which of the three subtypes of DE is present^40^: aqueous-deficient DE, decreased-wettability DE, and increased-evaporation DE, and each subtype should be examined separately. However, due to the lack of cases, we were not able to do so. Therefore, this is an issue that should be examined in studies with more cases.

## Conclusion

CAPs in tear fluid may be associated with MGD and DE, possibly related to alternative pathways. Since CAPs in tear fluid is associated with MGD and DE, anti-complement drugs could be new targets for MGD and DE treatment.

## Data Availability

The datasets used and/or analysed during the current study available from the corresponding author on reasonable request.

## References

[1] Craig JP, Nichols KK, Akpek EK, Caffery B, Dua HS, Joo CK (2017). TFOS DEWSII definition and classification report. Ocul. Surf..

[2] Tsubota K, Yokoi N, Watanabe H, Murat D, Kojima T, Yamada M (2020). A new perspective on dry eye classification: Proposal by the dry eye society. Eye Contact Lens.

[3] Arita R, Mizoguchi T, Kawashima M, Fukuoka S, Koh S, Shirakawa R (2019). Meibomian gland dysfunction and dry eye are similar but different based on a population-based study: The Hirado-Takushima study in Japan. Am. J. Ophthalmol..

[4] Dana R, Bradley JL, Guerin A, Pivneva I, Stillman I, Evans AM (2019). Estimated prevalence and incidence of dry eye disease based on coding analysis of a large, all-age United States health care system. Am. J. Ophthalmol..

[5] Song P, Xia W, Wang M, Chang X, Wang J, Jin S (2018). Variations of dry eye disease prevalence by age, sex and geographic characteristics in China: A systematic review and meta-analysis. J. Glob. Health.

[6] Foulks GN, Bron AJ (2003). Meibomiam gland dysfunction: a clinical scheme for description, diagnosis, classification, and grading. Ocul. Surf..

[7] Bron AJ, Tiffany JM (2004). The contribution of meibomian disease to dry eye. Ocul. Surf..

[8] Lam H, Bleiden L, Paiva CS, Farley W, Stern ME, Pelugfelder SC (2009). Tear cytokine profiles in dysfunctional tear syndrome. Am. J. Ophthalmol..

[9] Roda M, Corazza I, Reggiani M, Pellegrini M, Taroni L, Giannaccare G (2020). Dry eye disease and tear cytokine levels—A meta-analysis. Int. J. Mol. Sci..

[10] Fujita T, Matsushita M, Endo Y (2004). The lectin-complement pathway—Its role in innate immunity and evolution. Immunol. Rev..

[11] Bjork J, Hugli TE, Smedegard G (1985). Microvascular effects of anaphylatoxins C3a and C5a. J. Immunol..

[12] Foreman KE, Vaporciyan AA, Bonish BK (1994). C5a induced expression of P-selectin in endothelial cells. J. Clin. Investig.

[13] Crowley MA, Delgado M, Will Orrego A, Buchannan NM, Anderson K, Jaffee BD (2018). Induction of ocular complement activation by inflammatory stimuli and intraocular inhibition of complement factor D in animal models. Investig. Ophthalmol. Vis. Sci..

[14] Mondino BJ (1988). Inflammatory diseases of the peripheral cornea. Ophthalmology.

[15] Klein RJ, Zeiss C, Chew EY (2005). Complement factor J polymorphism in age-related macular degeneration. Science.

[16] Hageman GS, Anderson DH, Johnson LV, Hancox LS, Taiber AJ, Hardisty LI (2005). A common haplotype in the complement regulatory gene factor H(HF1/CFH) predisposes individuals to age-related macular degeneration. Proc. Natl. Acad. Sci. USA.

[17] Kato Y, Oguchi Y, Omori T, Shintake H, Tomita R, Kasai A (2020). Complement activation products and cytokines in pachychoroid neovasculopathy and neovascular age-related macular degeneration. Investig. Ophthalmol. Vis. Sci..

[18] Zaidi TS, Zaidi T, Pier GB (2010). Role of neutrophils, MyD88-mediated neutrophil recruitment, and complement in antibody mediated defense against Pseudomonas aeruginosa keratitis. Investig. Ophthalmol. Vis. Sci..

[19] Ballow M, Donshik PC, Mendelson L (1985). Complement proteins and C3 anaphylatoxin in the tears of patients with conjunctivitis. J. Allergy Clin. Immunol..

[20] Imanishi J, Takahashi F, Inatomi A, Tagami H, Yoshikawa T, Kondo M (1982). Complement levels in human tears. Jpn. J. Ophthalmol..

[21] Yazdani M, Benedikte K, Rootwelt H, Shahdadfar A, Utheim P, Utheim TP (2019). Tear metabolomics in dry eye disease: A review. Int. J. Mol. Sci..

[22] Maehara H, Norikawa K, Tanaka K, Kato Y, Kasai A (2023). Tear fluid and complement activation products in tears after ocular surgery. BMC Ophthalmol..

[23] Koh S, Inoue Y, Ochi S, Takai Y, Maeda N, Nishida K (2017). Quality of vision in eyes with epiphora undergoing lacrimal passage intubation. Am. J. Ophthalmol..

[24] Shimazaki J, Yokoi N, Watanabe H, Amano S, Ohashi Y, Kinoshita S (2017). Definition and diagnosis of dry eye in Japan, 2016. Atarashii Ganka (J. Eye).

[25] Nichols KK, Foulks GN, Bron AJ, Glasgow BJ, Dogru M (2011). The international workshop on meibomian gland dysfunction: Executive summary. Investig. Ophthalmol. Vis. Sci..

[26] Schiffman RM, Christianson MD, Jacobsen G, Hirsch JD, Reis BL (2000). Reliability and validity of the ocular surface disease index. Arch. Ophthalmol..

[27] Zakaria N, Grasdorff SV, Wouters K, Rozema J, Koppen C, Lion E (2012). Human tears reveal insights into corneal neovascularization. PLoS One.

[28] Ricklin D, Reis ES, Mastellos DC, Gros P, Lambris JD (2016). Complement component C3—The “Swiss Army Knife” of innate immunity and host defense. Immunol. Rev..

[29] Imagawa DK, Osifchin NE, Paznekas WA (1983). Consequences of cell membrane attack by complement: Release of arachidonate and formation of inflammatory derivatives. Proc. Natl. Acad. Sci. USA.

[30] Choi M, Han SJ, Ji YW, Choi YJ, Jun I, Alotaibi MH (2019). Meibum expressibility improvement as a therapeutic target of intense pulsed light treatment in meibomian gland dysfunction and its association with tear inflammatory cytokines. Sci. Rep..

[31] Chen X, Rao J, Zheng Z, Yu Y, Lou S, Liu L (2019). Integrated tear proteome and metabolome reveal panels of inflammatory-related molecules via key regulatory pathways in dry eye syndrome. J. Proteom. Res..

[32] Yoon KC, Jeong IY, Park YG, Yang SY (2007). Interleukin-6 and tumor necrosis factor-α levels in tears of patients with dry eye syndrome. Cornea.

[33] Willems B, Tong L, Minh TD, Pham ND, Hiep X, Zumbansen M (2021). Novel cytokine multiplex assay for tear fluid analysis in Sjögren’s syndrome. Ocul. Immunol. Inflamm..

[34] Tsuru H, Osaka M, Hiraoka Y, Yoshida M (2020). HFD-induced hepatic lipid accumulation and inflammation are decreased in factor D deficient mouse. Sci. Rep..

[35] Ito S, Hasimoto H, Yamakawa H, Kusumoto D, Akiba Y, Nakamura T (2022). The complement C3-complement factor D-C3a receptor signalling axis regulates cardiac remodelling in right venticular failure. Nat. Commun..

[36] Nichols KK, Foulks GN, Bron AJ, Glasgow BJ, Dogru M, Tsubota K (2011). The international workshop on meibomian gland dysfunction: Executive summary. Investig. Ophthalmol. Vis. Sci..

[37] Luo L, Li DQ, Doshi A, Farley W, Corrales RM, Pflugfeleder SC (2004). Experimental dry eye stimulates production of inflammatory cytokines and MMP-9 and activates MARK signaling pathways on the ocular surface. Investig. Ophthalmol. Vis. Sci..

[38] Brodsky R (2008). Advances in the diagnosis and therapy of paroxysmal nocturnal hemoglobinuria. Blood.

[39] Sciascia S, Radin M, Yazdany J, Tektoidou M, Cecchi I, Roccatello D (2017). Expanding the therapeutic options for renal involvement in lupus: Eculizumab, available evidence. Rheumatol. Int..

[40] Yokoi N, Georgiev GA, Kato H, Komuro A, Sonomura Y, Sotozono C (2017). Classification of fluorescein breakup patterns: A novel method of differential diagnosis for dry eye. Am. J. Ophthalmol..

